# Electro-Optical Properties of Monolayer and Bilayer Pentagonal BN: First Principles Study

**DOI:** 10.3390/nano10030440

**Published:** 2020-02-29

**Authors:** Mehran Amiri, Javad Beheshtian, Farzaneh Shayeganfar, Mahdi Faghihnasiri, Rouzbeh Shahsavari, Ali Ramazani

**Affiliations:** 1 Department of Chemistry, Faculty of Science, Shahid Rajaee Teacher Training University, 16788-15811 Tehran, Iran; mehranamiri92@gmail.com (M.A.); mahdi.faghihnasiri@gmail.com (M.F.); 2 Department of Physics and Energy Engineering, Amirkabir University of Technology, 15916-39675 Tehran, Iran; 3 Department of Civil and Environmental Engineering, Rice University, Houston, TX 77005, USA; rouzbeh@rice.edu; 4 Department of Mechanical Engineering, MIT, Cambridge, MA 02139, USA; ramazani@mit.edu

**Keywords:** mono/bilayer pentagonal BN, metallic behavior, optical properties, electronic properties

## Abstract

Two-dimensional hexagonal boron nitride (hBN) is an insulator with polar covalent B-N bonds. Monolayer and bilayer pentagonal BN emerge as an optoelectronic material, which can be used in photo-based devices such as photodetectors and photocatalysis. Herein, we implement spin polarized electron density calculations to extract electronic/optical properties of mono- and bilayer pentagonal BN structures, labeled as $B_{2}N_{4}$, $B_{3}N_{3}$, and $B_{4}N_{2}$. Unlike the insulating hBN, the pentagonal BN exhibits metallic or semiconducting behavior, depending on the detailed pentagonal structures. The origin of the metallicity is attributed to the delocalized boron (B) 2p electrons, which has been verified by electron localized function and electronic band structure as well as density of states. Interestingly, all 3D networks of different bilayer pentagonal BN are dynamically stable unlike 2D structures, whose monolayer $B_{4}N_{2}$ is unstable. These 3D materials retain their metallic and semiconductor nature. Our findings of the optical properties indicate that pentagonal BN has a visible absorption peak that is suitable for photovoltaic application. Metallic behavior of pentagonal BN has a particular potential for thin-film based devices and nanomaterial engineering.

## 1. Introduction

Boron nitride (BN) is a two-dimensional (2D) nanomaterial with great similarity to graphene, sometimes called white graphene, which is insulator-like with a wide band gap [1,2,3,4]. BN compounds possess exceptional mechanical [1], optical [3,4,5], catalytic [6], and thermal [7] properties comparable to graphene and carbon based nanocomposites. Therefore, BN is used for a wide range of industrial application with harsh environments such as high temperature ceramic composites [8,9]. All BN including wurtzite BN, cubic-BN, hexagonal BN (h-BN), and BN polymorphs [10,11,12,13,14,15] are insulators and remain insulating under high level compression [8]. More recently, Zhang et al. [8] discovered that three-dimensional (3D) tetragonal BN becomes metallic, which is dynamically a stable phase.

One-dimensional (1D) BN nanotubes are insulators [16,17,18,19], while 2D BN such as BN quantumdot are semiconductors [20]. However, carbon nanotubes could be metallic or semiconducting depending on their geometry, chirality, and radius [21,22,23]. Several attempts have been done to engineer 2D BN materials, tune their electronic and optical properties, with applications in nanoelectronic devices as well as photovoltaic arrays [24,25,26]. For instance, Lopez-Bezanilla et al. [26] reported that the O functionalized BN nanoribbons are metallic and show ferrimagnetic character. Moreover, half metallicity of BN nanoribbons has been observed by Barone and Peralta [27]. Several groups reported that the B-edge terminated BN nanoribbons with H atoms [28] and F atoms [25] show half metallicity. We recently reported that the edge functionalized BNQD and side defect BNQD behave as a semiconductor with quantum emission in a visible region [29].

A challenge for practical application of BN compound is related to its wide band gap. This feature can be manipulated by using some strategies for band gap closing such as doping, applying an external electric field, applying strain, and stress [25,26,30]. Seeking pentagonal BN sheets has been inspired by a recent report of the existence of penta-graphene as a new carbon allotrope, indicating ultrahigh strength and opening a 3.25 eV band gap. For 2D hBN, in a plane polar covalent bond between B, N atoms break the bipartite honeycombs symmetry and create a wide band gap.

In a 3D BN, Zhang et al. [8] discovered that a 3D BN with interlocking hexagons becomes metallic due to the coexistence of hybrid $sp^{2}$ and $sp^{3}$ bands. Zeng et al. [31] experimentally investigated the conductivity of BN nanoribbons by unwrapping multiwalled BN nanotubes through plasma etching. The origin of conductivity of BN nanoribbons was attributed to vacancy defects and bare edges.

Herein, we study the possibility of designing 2D BN nanosheets, possessing intrinsic metallicity without applying external agents or functionalization parameters. We investigate the electro-optical properties of 2D monolayer and bilayer pentagonal BN with the help of a first-principles study. We show that some pentagonal BN structures become metallic by geometry engineering. Bilayer pentagonal BN is stable due to an interfacial effect and exhibits both metallic and semiconductor behavior. Optical properties of monolayer and bilayer pentagonal BN reveal that metallic and semiconducting states of these pentagonal BN structures can emit and adsorb visible light, suitable for photovoltaic arrays. This new class of monolayer and bilayer pentagonal BN provides a pathway to achieve metallic BN.

## 2. Methods

**Geometry and electronic structures.** To study the geometry stabilization and electronic properties of monolayer and bilayer pentagonal BN structures, we carried out quantum computation based on density functional theory (DFT), implemented in a Vienna Ab initio Simulation Package (VASP) [32,33,34]. To describe the exchange interaction, we use the generalized gradient approximation (GGA) with a Perdew–Burke–Ernzerhof (PBE) ad to treat ion–electron interaction the projector-augmented wave (PAW) potential applied with an energy cutoff of 500 eV.

For bilayer structures, the vdW correction DFT-D2 is applied to take into account the interaction between BN layers. To prevent the interaction between periodic cells, a vacuum space of 20 Å is used [35]. All of the atoms in monolayer and bilayer structures were relaxed with a force convergence of 10${}^{-2}$ eV/Å and a total energy convergence of $10^{-5}$ eV. The k-sampling for the first Brillouin zone was 15×15×1 within the Monkhorst–Pack sampling k-mesh for the geometry optimization.

It is worth noting that the interaction energy of a pentagonal BN layer in bilayer structure is computed by:

(1) $$E_{int}=E_{tot}\left(bilayer\right)-2E_{tot}\left(monolayer\right)$$

where $E_{tot}\left(bilayer\right)$ is the total energy of bilayer pentagoal BN and $E_{tot}\left(monolayer\right)$ is the total energy of monolayer pentagonal BN.

**Phonon spectrum**. Lattice phonon spectra are calculated using the density functional theory and finite displacement method as implemented in VASP and phonopy [32,33,34]. The energy convergence for the phonon spectra were set to $10^{-8}$ eV for total energy and 0.01 eV/Å for force.

**Optical properties.** To determine the optical properties of materials, we calculated the dielectric function, which represents linear behavior of materials subjected to the electromagnetic radiation. The complex dielectric function consists of real and imaginary parts defined as:

(2) $$\epsilon_{complex}=ℜ\left(\epsilon \left(\omega \right)\right)+iℑ\left(\epsilon \left(\omega \right)\right)=\epsilon_{1}\left(\omega \right)+i\epsilon_{2}\left(\omega \right)$$

The real part $\epsilon_{1}$ describes the polarization and the imaginary part $\epsilon_{2}$ the absorption of materials. The imaginary part $\epsilon_{2}$ is calculated using the random phase approximation: [36]

(3) $$ℑ\epsilon_{\alpha }\left(\omega \right)=\frac{4\pi^{2}e^{2}}{m^{2}\omega^{2}}\Sigma_{i,j}\int \frac{2dk^{3}}{2\pi^{3}}{<ik|p_{\alpha }|fk>}^{2}f_{i}^{k}\left(1-f_{f}^{k}\right)\delta \left(E_{f}^{k}-E_{i}^{k}-\hbar \omega \right)$$

where α index represents the light polarizability, and *i* and *f* indicate initial and final states, respectively. This equation represents the contribution of the interband transition [37], where |ik> represents the state vector for the initial position, |fk> represents the state vector for the final position, $f_{i}^{k}$ and $f_{f}^{k}$ represent the Fermi distribution function of occupied and unoccupied states, and $p_{\alpha }$ is the momentum operator, respectively. Gaussian broadening of 0.1 eV is applied to the dielectric function calculations. A highly dense k-mesh, 24×24×1, is used to reach an accuracy suitable to calculate the optical spectra [38,39,40].

The optical absorption coefficient, α(ω), is defined as: [41]

(4) $$\alpha \left(\omega \right)=2\omega \sqrt{1/2[-\epsilon_{1}\left(\omega \right)+\sqrt{\epsilon_{1}{\left(\omega \right)}^{2}+\epsilon_{2}{\left(\omega \right)}^{2}}]}$$

where ω has units of energy (in atomic units).

## 3. Results

### 3.1. Monolayer Pentagonal BN

**Figure 1:**
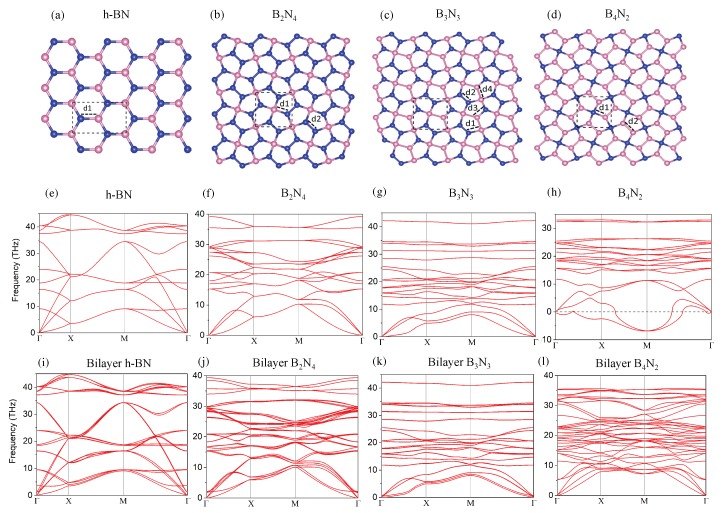
(**a**–**d**) unitcell of atomic configuration of monolayer pentagonal BN; (**e**–**h**) phonon spectra of monolayer pentagonal BN; (**i**–**l**) phonon spectrum for bilayer pentagonal BN.

**Atomic Configuration**—We designed the 2D pentagonal BN structures, which have three different unit cells as shown in Figure 1b–d, as $B_{2}N_{4}$, $B_{3}N_{3}$, and $B_{4}N_{2}$. The structural parameters of optimized monolayer pentagonal BN are presented in Table 1 via interatomic nearest neighbors ($d_{1}$, $d_{2}$, $d_{3}$, $d_{4}$), as shown in Figure 1a–d.

To ensure the stability of the different pentagonal BN structures, we computed the phonon spectra, which is plotted in Figure 1f–h. $B_{4}N_{2}$ has negative phonon bands, which is unstable in close agreement with previous reports [35]. However, for bilayer pentagonal BN (Figure 1j–l), all phonon modes are positive and real, and there are no negative and imaginary modes in the Brillouin zone, confirming the dynamical stable structures.

The vibrational modes in the band dispersion are separated into low and high frequency regions by phonon gap or stop bands. The in-plane modes, both transverse and longitudinal, show linear dispersion near the Γ point, while the out of plane modes indicate quadratic dispersion. This detailed analysis is reminiscent of some common features of 2D materials.

To check thermal stability of these BN allotropes, we carried out the DFT+MD for optimized structures, which support the thermal stability of our structures up to 400 K.

**Figure 2:**
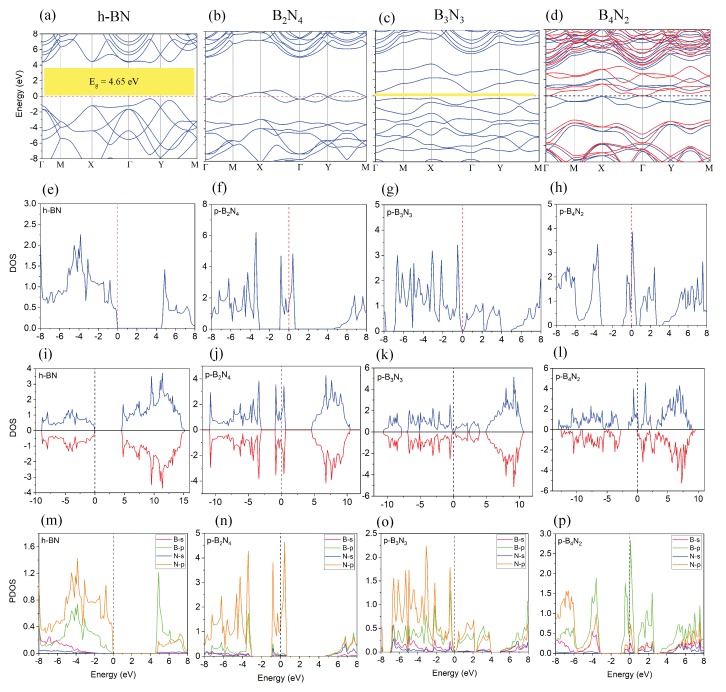
(**a**–**d**) electronic band structure of the h-BN and monolayer pentagonal BN; (**e**–**h**) total electronic density of states (DOS) and (**i**–**l**) total spin polarized DOS of the h-BN and monolayer pentagonal BN; (**m**–**p**) projected density of states (PDOS) for bilayer pentagonal BN.

**Electronic Properties**—After investigation of dynamical stability, we moved to study electronic properties of pentagonal BN structures by calculating the electronic band structure. The band structure results are shown in Figure 2 for different pentagonal BN compared to h-BN. We see that h-BN (Figure 2a) has a wide band gap of 5 eV, in good agreement with experimental and theoretical results [42]. Occupied electronic states in the vicinity of Fermi level in Figure 2b,d manifest the metallicity of $B_{2}N_{4}$, $B_{4}N_{2}$ structures. However, Figure 2c confirms a band gap of 0.11 eV for $B_{3}N_{3}$, suggesting a band gap closing relative to that of h-BN.

To discover the origin of semiconducting and metallicity in pentagonal BN, we computed the total electronic density of states (DOS) as shown in Figure 2e–h and Figure 2i–l. We note that spin polarized electron density in Figure 2l shows different spin up and down electron density, confirming magnetic moment (1.07) for pentagonal $B_{4}N_{2}$ structure. The magnetic property is originated from the unstable nature of $B_{4}N_{2}$ structure, which causes spin population for up states and down states to become different and create magnetic moments in the structure.

**Figure 3:**
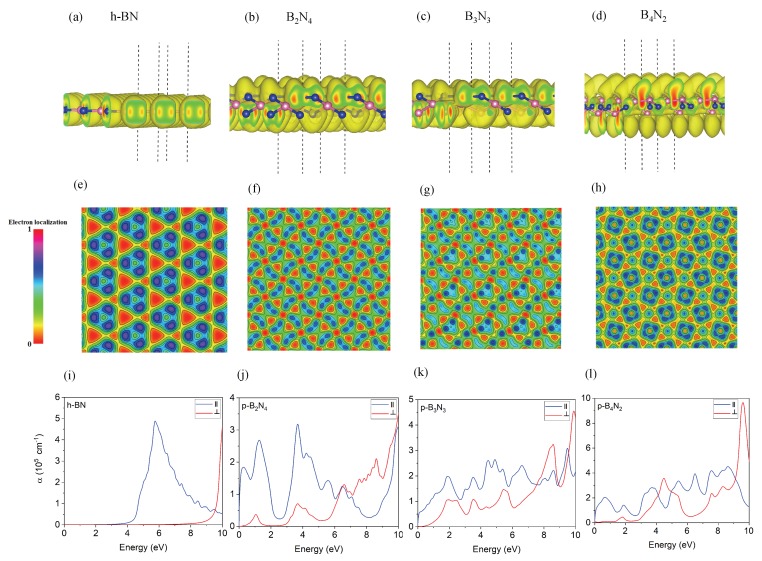
(**a**–**d**) the electron localization function (ELF) with hybridization orbitals of the monolayer h-BN and monolayer pentagonal BN; (**e**–**h**) the ELF of the monolayer pentagonal BN compared with h-BN. The reference bar for ELF brought at the right with isovalue 0.15 e/A${}^{3}$ for charge density; (**i**–**l**) optical absorption spectrum of monolayer h-BN and monolayer pentagonal BN.

To find out the origin of metallicity of pentagonal $B_{2}N_{4}$, and $B_{4}N_{2}$ structures, we analyzed the electron localization function (ELF) in Figure 3. ELF map describes the electron delocalization of solids [43] and liquids [44] as an analytical tool for characterization of the chemical bond. [45] The ELF map displays a theory of jellium like homogeneous electron gas, renormalizing the isosurface values between 0.0 and 1.0. The fully delocalized electrons are represented by 0.5 and fully localized electrons by 1, while value 0.0 indicates low level charge density. These ELF figures show that the charge density around boron atoms in Figure 3b,d,f,h contributed to delocalize electrons (blue colors), while red colors represent the localized electron density. Therefore, a conducting electron network of $B_{2}N_{4}$, and $B_{4}N_{2}$ is created, governing the metallic behavior of pentagonal BN.

**Optical Properties**—To investigate optical absorption spectrum, we compute real and imaginary part of dielectric function ($\epsilon \left(\omega \right)=\epsilon_{1}\left(\omega \right)+i\epsilon_{2}\left(\omega \right)$) as described in the Methods section. Figure 3i–l indicated the absorption spectra α for all monolayer pentagonal structures including h-BN.

For h-BN, the first peak appears in the invisible range of 6 eV, while monolayer pentagonal BN structures show several peaks in the visible region. An ability to modify the optical properties and absorption spectra are important for devices such as photovoltaic and solar cells.

Regarding $B_{2}N_{4}$, the main parallel polarized peak is located at 0.2 eV and 1.9 eV with high intensity, while other peaks are located in the UV region. However, the main peak of $B_{3}N_{3}$ is located at 2 eV with intensity lower than that of $B_{2}N_{4}$, and, finally, two main parallel polarized peaks of $B_{4}N_{2}$ are located at 0.75 eV and 2 eV. These optical findings support our electronic results, which suggest that $B_{4}N_{2}$ has the zero band gap, but is unstable, while $B_{2}N_{4}$ has a zero band gap and is stable.

### 3.2. Bilayer Pentagonal BN

**Figure 4:**
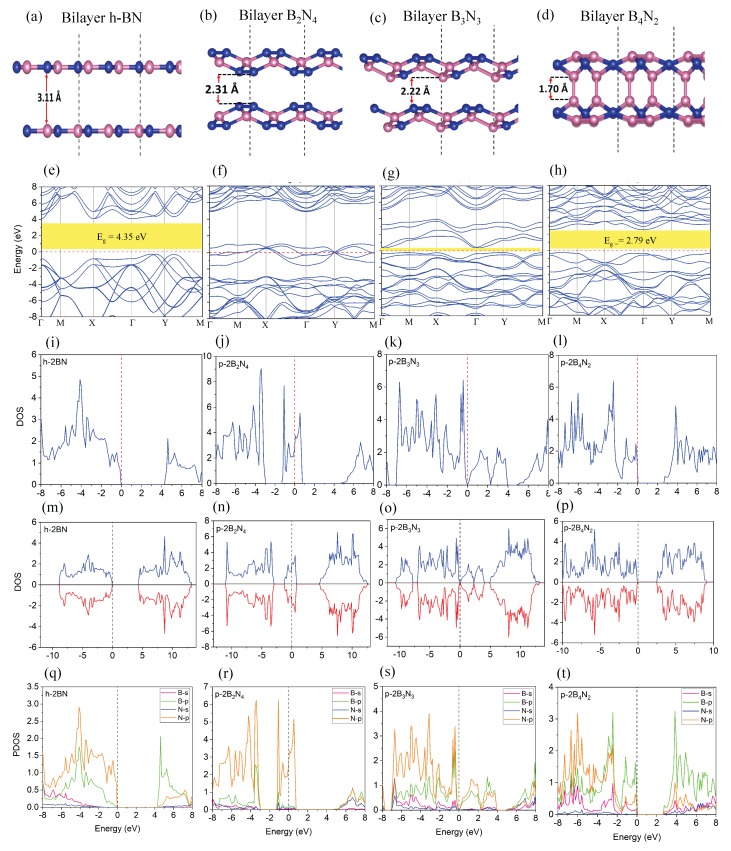
(**a**–**d**) unitcell of atomic configuration of bilayer pentagonal BN, (**e**–**h**) electronic band structure of the h-BN and monolayer pentagonal BN; (**i**–**l**) total electronic density of states (DOS) and (**m**–**p**) total spin polarized DOS of the h-BN and monolayer pentagonal BN; (**q**–**t**) projected density of states (PDOS) for bilayer pentagonal BN.

Modulating the electronic properties of BN by geometry engineering, we now extend our study to bilayer pentagonal BN, termed as bilayer $B_{2}N_{4}$, $B_{3}N_{3}$, and $B_{4}N_{2}$, and their unicells are plotted in Figure 4a–d.

**Atomic Configuration and Dynamical stability**—To confirm dynamical stability of bilayer pentagonal BN, we calculated phonon spectra. As shown in Figure 1i–l, bilayer pentagonal BN structures have no imaginary modes, confirming dynamical stability. The interaction energy and total energy of all monolayer and bilayer pentagonal BN studied in this work are presented in Table 2.

DFT results indicate that the interaction energy for bilayer $B_{4}N_{2}$ is −8.44 eV, which is greater than the two other bilayer structures. This interlayer interaction of bilayer $B_{4}N_{2}$ creates a quite stable structure without negative modes as can be inferred from Figure 1l.

**Figure 5:**
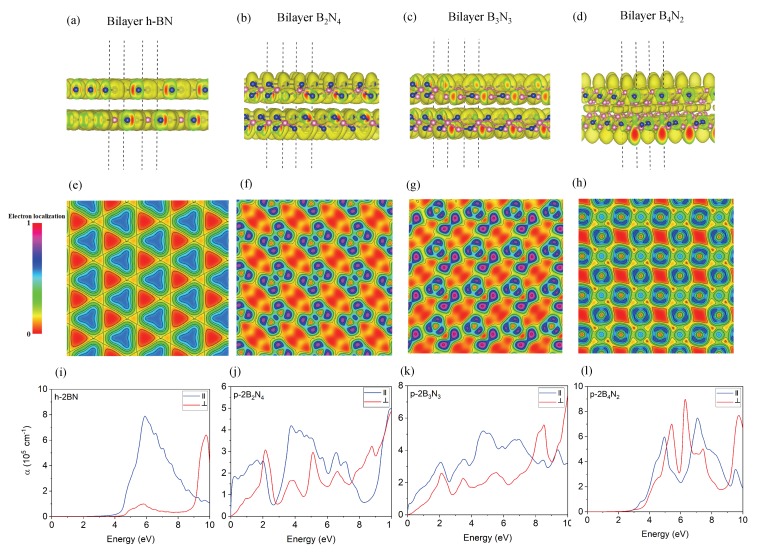
(**a**–**d**) the electron localization function (ELF) with hybridization orbitals of the bilayer h-BN and bilayer pentagonal BN; (**e**–**h**) the ELF of the bilayer pentagonal BN compared with bilayer h-BN. The reference bar for ELF brought at the right with isovalue 0.15 e/A${}^{3}$ for charge density; (**i**–**l**) optical absorption spectrum of bilayer h-BN and bilayer pentagonal BN.

**Electronic properties**—The electronic property calculations on bilayer pentagonal BN are plotted in Figure 4, which reveal that the bilayer $B_{2}N_{4}$ is metallic with no band gap (Figure 4f), whereas the other bilayer pentagonal BNs are semiconducting (Figure 4g,h) with band gaps of 0.092 eV and 2.79 eV, respectively (Table 2). Further analysis of electronic band structures of Figure 4d manifest that there is a band gap opening for bilayer pentagonal $B_{4}N_{2}$ related to strong interaction of bilayer pentagonal BN, while its monolayer has no band gap. This strong interaction between layers creates localized electrons (Figure 4t, boron p orbitals) and electrical dipole moments, from which semiconducting behavior emerges. The total DOS and spin polarized DOS are calculated and plotted in Figure 4i–p. These DOSs reveal more electron states contributed from the interlayer states due to orbital hybridization and mixing orbitals. Moreover, Figure 4n–p manifest that there is no magnetic moment for bilayer pentagonal BN. The ELF maps in Figure 5a–h indicate $sp^{2}-sp^{3}$ hybridized of bilayer pentagonal structures, supporting interaction between the layers (Figure 5a–h).

**Optical properties**—Figure 5i–l shows the absorption spectra for bilayer h-BN and pentagonal BN. Analysis of Figure 5i indicates that the main peak of absorption spectrum of bilayer hBN is placed at 6 eV, confirming its insulating nature. However, Figure 5j shows a broad parallel polarized spectrum in visible range (0–2.5 eV) for bilayer pentagonal $B_{2}N_{4}$, which is metallic. Spectral analysis of Figure 5k for bilayer pentagonal $B_{3}N_{3}$ states that the first parallel polarized peak is located at 2.1 eV in a visible range, while bilayer pentagonal $B_{4}N_{2}$ has a weak parallel polarized peak in the visible region located at 3.2 eV and supports the semiconducting nature of $B_{4}N_{2}$. These optical results are in line with our calculated electronic properties.

## 4. Discussion

Our study shows that the monolayer pentagonal $B_{2}N_{4}$ and $B_{4}N_{2}$ exhibit a metallic behavior. To fully understand the origin of metallicity in these structures, we recall that boron has a multielectron bonds related to its electron deficient nature [26,46,47].

Previous study discussed that magnesium diboride $MgB_{2}$ is a superconductor, where Mg atoms are encapsulated in B layers [46,47]. In the $MgB_{2}$ case, a 2p state of B atoms creates delocalized π electron states, leading to metallicity and superconductivity, which is due to the π states correlations with the in-plane vibrational electrons of boron [46,47].

Zhang et al. [8] proposed that the tetragonal phase of $B_{3}N_{3}$ is metallic, which stems from the electron delocalization of cyclic borazine (B3N3H6) [48,49]. They discussed that borazine as an inorganic specimen of benzene has similar properties as benzene, including delocalized π electrons [48,49].

Recently, some experimental studies revealed that elements such as boron and hydrogen can lead to insulator–metal transition [50], while other elements such as sodium and lithium can undergo metal–insulator transition by structural deformation due to compression [51,52,53].

In this study, we demonstrated for the first time that 2D BN become metallic, when the atomic geometry is modified. Our results decode the structural and geometrical effect of BN thin films.

## 5. Conclusions

We investigated the electronic and optical properties of three polymorphs of monolayer and bilayer pentagonal BN, namely $B_{2}N_{4}$, $B_{3}N_{3}$, and $B_{4}N_{2}$, using spin polarized electron density calculation. We examined the electronic band structure, total/spin-polarized-projected DOS, and optical absorption spectrum. We found that the monolayer pentagonal $B_{4}N_{2}$ exhibits metallic behavior, and is unstable with negative phonon bands, while bilayer pentagonal $B_{4}N_{2}$ exhibits a semiconducting nature and is energetically stable. However, bilayer pentagonal BN generally shows both metallic and insulating behavior, depending on the atomic arrangement. For instance, bilayer pentagonal $B_{2}N_{4}$ demonstrates a metallic nature, while bilayer pentagonal $B_{3}N_{3}$ and $B_{4}N_{2}$ show a semiconducting nature with the band gap of 0.09 eV and 2.79 eV.

Furthermore, the optical absorption spectra of the monolayer pentagonal BN show several peaks in the visible range, making them a good candidate for photovoltaic applications.

Overall, our results demonstrate that monolayer and bilayer pentagonal BN polymorphs and their tunable electronic and optical properties are applicable to photodetectors, photovoltaic, and photocatalysis.

## Figures and Tables

**Table 1:** The structural parametere or bond length ($d_{1}$, $d_{2}$, $d_{3}$, $d_{4}$) of optimized monolayer pentagonal BN as shown in Figure 1a–d.

	Lattice (Å)	$d_{1}$	$d_{2}$	$d_{3}$	$d_{4}$
h−BN	a = 4.35, b = 2.51	1.45	—	—	—
$B_{2}N_{4}$	a = b = 3.62	1.55	1.34	—	—
$B_{3}N_{3}$	a = b = 3.75	1.60	1.34	1.34	1.78
$B_{4}N_{2}$	a = b = 3.79	1.57	1.59	—	—

**Table 2:** Electronic properties of monolayer and bilayer pentagonal BN structures.

	Energy	Interaction Energy	Fermi Energy	Band Gap
h−BNmonolayer (eV)	−35.45	—	−4.48	4.65
h−BNbilayer (eV)	−71.19	−0.27	−3.16	4.35
$B_{2}N_{4}\;monolayer$ (eV)	−47.96	—	−3.33	0
$B_{2}N_{4}\;bilayer$ (eV)	−96.35	−0.43	−1.51	0
$B_{3}N_{3}\;monolayer$ (eV)	−45.41	—	−3.80	0.11
$B_{3}N_{3}\;bilayer$ (eV)	−91.20	−0.37	−2.11	0.092
$B_{4}N_{2}\;monolayer$ (eV)	−41.43	—	−2.05	0
$B_{4}N_{2}\;bilayer$ (eV)	−91.31	−8.44	−1.77	2.79
